# How are progression decisions made following external randomised pilot trials? A qualitative interview study and framework analysis

**DOI:** 10.1186/s13063-022-06063-9

**Published:** 2022-02-10

**Authors:** Katie Mellor, Susan J. Dutton, Sally Hopewell, Charlotte Albury

**Affiliations:** 1 Oxford Clinical Trials Research Unit/Centre for Statistics in Medicine, Nuffield Department of Orthopaedics, Rheumatology and Musculoskeletal Sciences, University of Oxford, Oxford, England, UK; 2 Nuffield Department of Primary Care Health Sciences, University of Oxford, Oxford, England, UK

**Keywords:** Qualitative research, Pilot trials, Randomised controlled trials, Feasibility studies, Progression criteria

## Abstract

**Background:**

External randomised pilot trials help researchers decide whether, and how, to do a future definitive randomised trial. The progression criteria are often prespecified to inform the interpretation of pilot trial findings and subsequent progression decision-making. We aimed to explore and understand the perspectives and experiences of key stakeholders when making progression decisions following external pilot trials.

**Methods:**

Thirty-five remote semi-structured interviews with external randomised pilot trial team members including chief investigators, trial managers, statisticians and patient and public involvement (PPI) representatives. Questions focussed on experiences and perceptions of pilot trial progression decisions and whether and how progression criteria informed this decision. Data were analysed using the framework method approach to thematic analysis. Strategies to ensure trustworthiness and rigour were used.

**Results:**

Interviews were conducted between December 2020 and July 2021. Six descriptive themes were developed to capture the experiences and perspectives of participants. These were (1) divided opinions on the value and development of progression criteria, (2) (avoiding) the potential for personal interest to influence progression criteria and progression decision-making, (3) stakeholder engagement in setting progression criteria and making progression decisions, (4) lessons learned from doing the pilot trial and their impact on progression criteria applicability, (5) other factors that inform the progression decision and (6) progression of external randomised pilot trials—funding considerations and constraints. These themes were underpinned by an overarching interpretative theme ‘a one-size approach to progression does not fit all’ to describe the highly nuanced and complex decision-making process that occurs following external randomised pilot trials. The progression criteria are rarely the only consideration informing the decision to progress to future research; unanticipated events, signals of efficacy and continuity of the research team are other factors that researchers consider.

**Conclusions:**

One size does not fit all when it comes to the progression criteria and pilot trial progression. The progression criteria are only one of many considerations researchers have when deciding whether a pilot trial is feasible. External pilot trial progression is not guaranteed even when a pilot trial is considered feasible (based on the progression criteria and/or other considerations), indicating inefficiency and potential research waste.

**Trial registration:**

Open Science Framework osf.io/5N2KZ

**Supplementary Information:**

The online version contains supplementary material available at 10.1186/s13063-022-06063-9.

## Background

Randomised pilot trials aim to assess the feasibility of a definitive randomised controlled trial (RCT) where there are uncertainties about trial design [1]. The terms ‘pilot’ and ‘feasibility’ are used interchangeably in the literature. Since pilot trials are one type of feasibility study, they are often also legitimately referred to as ‘randomised feasibility studies’ [1]. Pilot trials inform the decision to progress to a definitive RCT and can be distinguished into two distinct groups: internal or external. Internal pilot trials are embedded within an RCT forming the first phase, for example, the first 6 months, where the feasibility of the RCT is being assessed whilst the RCT is being conducted [2]. External pilot trials are small stand-alone trials that are distinct from the definitive RCT that aims to assess whether a future planned RCT will be feasible [1]. For internal pilot trials, a decision to progress will mean proceeding directly into the main RCT phase of the study, where data collected during the internal pilot phase contributes to the main RCT dataset. For external pilot trials, a decision to progress will involve sourcing funding for the future RCT as a separate study, and data collected as part of the external pilot trial is not included in the final RCT analysis.

Prespecified progression criteria can inform progression decisions following pilot trials. Recommendations for the development and use of the progression criteria in the context of embedded internal pilot trials include agreeing criteria in advance with research funders, considering a green (go), amber (amend), red (stop) traffic light approach and involving funders and a Trial Steering Committee (TSC) in assessing the progression criteria [2]. There is, however, currently no evidence-based guidance or recommendations for best practice for using the progression criteria that is specific to external randomised pilot trials. In our previous study, we found that publications of external randomised pilot trials often do not report how the criteria are established, including who is involved, what rationale criteria are based on and how the criteria subsequently guide progression decisions [3]. This is important because the progression criteria aim to ensure that progression decision-making is unbiased, yet it is not always clear how they have been developed and will be interpreted. A lack of transparency could lead to misinterpretation of the criteria and inconsistency in the way that pilot trials are evaluated. This key issue regarding how stakeholders use progression criteria in external randomised pilot trials requires further exploration to understand the current practices in how the criteria are developed and interpreted to inform progression decision-making.

The aim of this study was to explore the experiences of key stakeholders in relation to external randomised pilot trial progression decision-making, including the development, application and assessment of the progression criteria. To meet this aim, we had the following objectives:

- To examine stakeholder views and experiences of developing, using and assessing progression criteria
- To identify additional factors which contribute to progression decision making
- To elicit barriers to using progression criteria in practice

Understanding how the progression criteria are currently being used in practice, including any barriers and additional factors that are considered when making progression decisions, is a crucial first step towards developing recommendations for best practice when using the progression criteria in external randomised pilot trials.

## Methods

We conducted remote semi-structured interviews to understand and examine the perspectives and experiences of key stakeholders in the United Kingdom (UK) who are involved in designing, conducting and analysing external pilot trials to make decisions about the feasibility of future RCTs. Participants included researchers with experience of conducting external randomised pilot trials (e.g. chief investigators, trialists, statisticians), patient and public involvement (PPI) representatives for pilot trials and researchers conducting pilot or feasibility trial methodology research.

We registered the protocol on the Open Science Framework: osf.io/5N2KZ [4]. Reporting follows the consolidated criteria for reporting qualitative research (COREQ) [5].

### Participant sampling, recruitment and informed consent

We used a theoretical sampling approach [6]. Sampling was iterative and responsive to themes that we were developing from the data. We aimed initially for a purposive sample, seeking for variation in roles and research experiences, to identify participants who were information-rich [6, 7]. As we noticed patterns in the data and started to develop themes, we sought to sample further based on what we were noticing. We recruited participants through the UK Clinical Research Collaboration (UKCRC) [8], Trials Methodology Research Partnership (TMRP) [9] and UK Trial Managers’ Network (UKTMN) [10] newsletters and email lists. We asked regional Research Design Services (RDS) [11] to share information with researchers, and we emailed the corresponding authors of influential publications directly. We also shared recruitment information on Twitter. We additionally used a snowball approach, asking participants identified through these recruitment methods to recommend other people to contact, for example, PPI members on their trials or other researchers in their institutions.

We recruited participants until thematic data saturation, defined as the point at which no new themes were being developed, and existing themes are well explored and described with certainty and confidence [12]. We sent participants a participant information sheet (PIS) at the point of arranging the interview and sought verbal informed consent before the interview started. Participants who were PPI representatives for pilot trials were offered a £20 One4All e-voucher.

### Data collection

Semi-structured interview guides were developed a priori by KM and CA and were commented on by a researcher with experience in conducting external randomised pilot trials. The interview guide for researchers explored participants’ experiences and perceptions of pilot trial progression decisions and whether and how the progression criteria informed this decision. Since the extent to which PPI representatives are involved in decisions around pilot trial progression is unknown, the interview guide for PPI representatives focussed on eliciting how, where and when they were involved in decisions around the progression criteria and progression decision-making. Following best practice [13], topic guides were flexible allowing participants to guide the conversation and present what was important to them. We also updated topic guides throughout data collection to capture unanticipated topics we wished to explore further. The final versions of both interview guides are presented in Additional file 1.

KM conducted all interviews. Interviews were semi-structured and took place on Microsoft Teams (Office365/Nexus365), lasting between 23:50 and 01:03:04 (average 44:31). Interviews were audio-recorded on two Olympus Dictaphones (DS-650 and LS-P1). KM took field notes after each interview detailing the reflexive and analytical thoughts that prompted changes to topic guides or the sampling strategy. KM’s ontological and epistemological positioning followed a pragmatist approach.

### Data analysis

Data analysis followed the seven stages of the framework method approach to thematic analysis [14]. The stages include transcription, familiarisation, coding, development of an analytical framework, application of the analytical framework, charting of data into framework matrices and interpretation of the data. We also incorporated aspects of reflexive thematic analysis [15], remaining cognisant of the role of the research team in co-creating data throughout data collection and analysis, and the importance of engaging in reflexivity and discussion throughout.

**Fig. 1:**
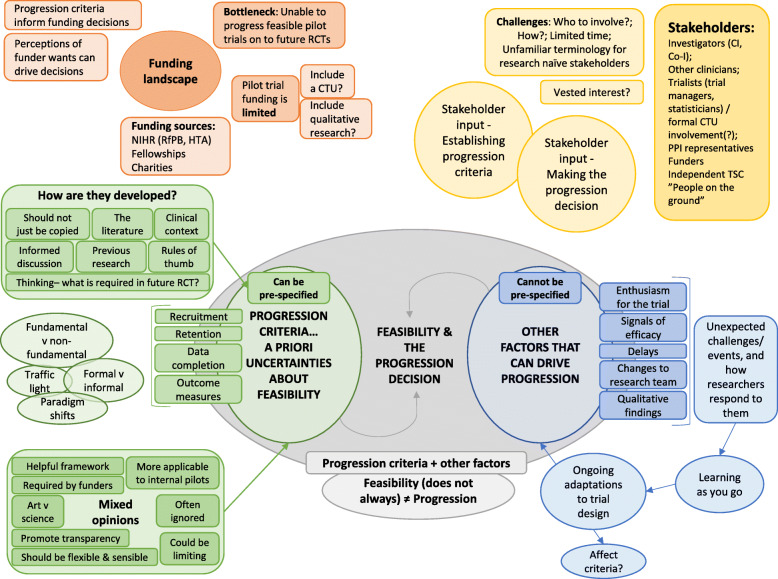
Visual map that was used to facilitate the development of themes

Data were transcribed verbatim, and then manged, coded and handled using NVivo 12. Analysis was led by KM, with input from CA, SH and SD. KM read each transcript and listened to the interview recording to support familiarisation. KM and CA coded the first two transcripts in full independently and engaged in peer discussion. They followed a line-by-line descriptive coding approach. Codes of similar content with shared properties and characteristics were grouped hierarchically into categories. KM applied this coding method to four further transcripts and developed the initial analytical framework, which was discussed with CA, and updated following discussion. The analytical framework was based on the codebook and was flexible and responsive to change, being iteratively updated as new codes were developed based on data collection and analysis. The analytical framework was charted into framework matrices within Microsoft Excel (Office16). These framework matrices facilitated mapping within and between participants or ‘cases’ to explore concepts and relationships and to develop themes and highlight key quotes. A visual map (final version presented in Fig. 1) was produced to summarise the developing themes and facilitate peer discussion.

To enhance rigour and trustworthiness, we followed Lincoln and Guba’s approach to evaluating the trustworthiness of qualitative research studies [16]. Additional file 2 details further the techniques we used to establish and maintain credibility, transferability, dependability and confirmability [16–18].

## Results

We interviewed thirty-five participants, affiliated to twelve UK universities between December 2020 and July 2021. Participants included chief investigators (CIs), trial managers, trial statisticians, trial methodologists, PPI representatives, senior researchers with trial oversight roles and one health economist. Participants often held multiple roles, and Table 1 illustrates the areas of overlap between trial roles.

**Table 1:** Characteristics of the study sample

Roles and affiliations of participants	Chief investigator	Trial statistician	Trial methodologist	Trial manager	Health economist	Senior researcher/trial oversight	PPI representative	Affiliation to journal^a^	Affiliation to funding panel^b^	Affiliation to RDS^c^
Chief investigator	**13**	1	1	0	0	2	0	13	9	2
Trial statistician		**7**	4	0	0	3	0	6	5	4
Trial methodologist			**8**	0	1	5	0	6	6	5
Trial manager				**6**	0	2	0	2	0	0
Health economist					**1**	1	0	1	1	1
Senior researcher/trial oversight						**10**	0	8	7	3
PPI representative							**6**	1	2	2
Affiliation to journal^a^								**24**	16	7
Affiliation to funding panel^b^									**18**	8
Affiliation to RDS^c^										**9**

Note that Table 1 demonstrates the overlap in participant roles. For example, six trial managers were interviewed, two were senior researchers or provided trial oversight, and two were or were previously affiliated to a journal, e.g. in an editorial or peer reviewer capacity

*RDS* Research Design Service

^a^Includes both current and previous affiliations in editorial or peer reviewer capacity

^b^Includes both current and previous affiliations as a formal panel member or peer reviewer of funding applications

^c^Includes both current and previous affiliations to an RDS

In many cases, participants reported that the primary reason they did an external randomised pilot trial was to assess the significant uncertainties about their definitive trial design. Some participants described feeling that the progression criteria for external pilot trials should be different to those for internal pilot trials since the pathway to definitive RCT is different. We identified variation in how people developed and implemented the progression criteria within their external randomised pilot trial, with many relying on additional factors to inform decisions.

**Fig. 2:**
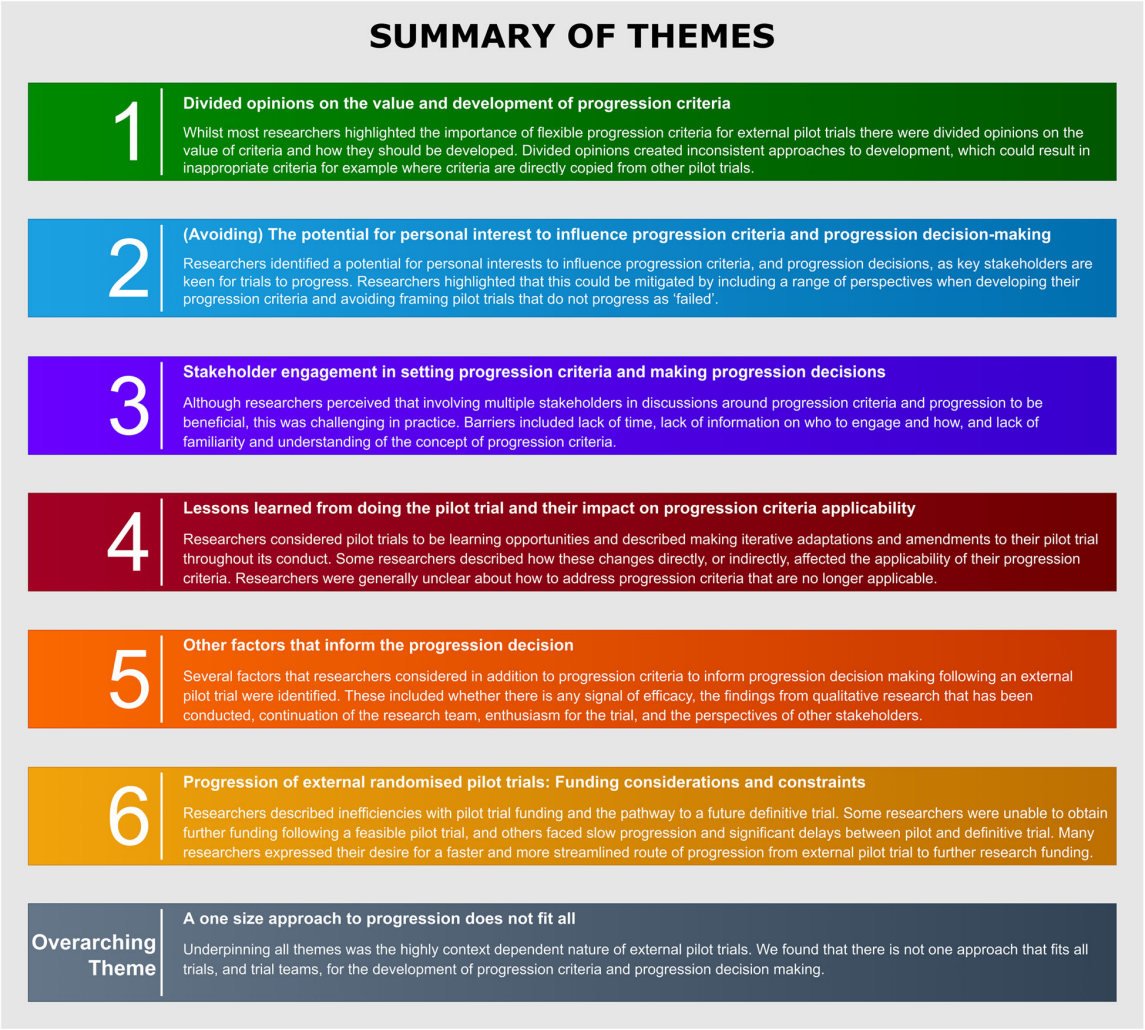
Summary of themes

We developed six descriptive themes around these results: (1) divided opinions on the value and development of progression criteria, (2) (avoiding) the potential for personal interest to influence the progression criteria and progression decision-making, (3) stakeholder engagement in setting the progression criteria and making progression decisions, (4) lessons learned from doing the pilot trial and their impact on progression criteria applicability, (5) other factors that inform the progression decision and (6) progression of external randomised pilot trials—funding considerations and constraints. We grouped by these descriptive themes rather than by the development, application and assessment of the progression criteria because views were similar across these different stages and our developed themes better presented our findings. To unite and explain these descriptive themes, we developed an overarching interpretive theme: a one-size approach to progression does not fit all. We present anonymised excerpts to illustrate and exemplify our findings and provide a short summary of each theme in Fig. 2.

### Theme 1: Divided opinions on the value and development of the progression criteria

Most participants were in consensus that pre-specifying how the pilot trial findings will be interpreted to draw conclusions about feasibility is important. However, there were mixed perceptions about whether the progression criteria are the best way to do this. Less experienced CIs who were conducting pilot trials as part of a fellowship described how the progression criteria provided a ‘helpful framework’ to consider uncertainties in their trial design and potential problems they might encounter:

I always have to remind myself when I’m running trials, if x happens, would I still be comfortable going forward with the trial? And if the answer is no then you have to have that as a progression criteria. [P021; CI]

Some researchers described that although they consider the progression criteria to be important and provide structure to their thinking, they are more of ‘an art than a science’; open to interpretation rather than being completely definitive:

I think you do need them, I think it’s necessary to kind of have something there so you’re not- to sort of structure your thinking… But at the same time it is a bit more of an art than a science thinking about how do you interpret those and how do you move kind of… beyond them? [P027; Senior researcher with trial oversight]

Some trial statisticians and senior researchers with trial oversight roles shared perceptions that the progression criteria were more appropriate to internal pilot trials, since there is often existing available data to base them on. One trial manager described not having formal progression criteria for their external pilot trial because progression is not guaranteed. Instead, they considered their progression criteria to be whether funding for further research could be obtained:

They were both external, so it wasn’t progression criteria because we weren’t progressing. The progression criteria is: Do we have any funding? [P006; Trial Manager]

Just as there were divided opinions in how helpful participants perceived progression criteria to be, there were also varied and inconsistent methods of criteria development. Divided opinions on ‘best practice’ could mean that the progression criteria were developed inappropriately. Participants described drawing on ‘rules of thumb’, observation work, previous research by their group or institution, published research, clinical experience, contextual considerations of what would be achievable in the future definitive trial or developing criteria through discussion. Experienced researchers recalled sharing examples of the progression criteria from previously successful funding applications with other researchers so they could consider what criteria may be appropriate for their pilot trial. However, other participants described their perceived tendency for researchers to directly copy the criteria, rather than adapting or changing them. Senior researchers discouraged this practice because it could result in mistakes being repeated rather than being learned from and that the same criteria were not likely to be applicable to different trial contexts. One CI described how the progression criteria they had developed for their pilot trial had been applied to different pilot trials which were in a completely different context:

There’s been quite a few where they’ve just literally taken the progression criteria that I’ve developed for this particular study and applied it to their own study, but it’s completely different […] in a completely different population. [P028; CI]

Although there were divided opinions in the value participants attributed to the progression criteria and in how they were developed, participants consistently emphasised the importance of flexibility and stated that the progression criteria should not be considered the *be-all and end-all*. Experienced researchers described two paradigm shifts in the way that they think about the progression criteria: (1) from initially not having any prespecified progression criteria, to having strict thresholds for progression *stop, go*; (2) to a more flexible approach, such as a traffic light or *stop, amend, go*. Some researchers stated that there can be variation in the traffic light approach, and it can be unclear what is meant by red, amber and green domains. One CI stated that including contingency plans in funding applications would be appropriate, but that word count limits could prohibit this:

If I were to take this to where I think I would be more comfortable is you have your green, amber, red criteria, but then you explicitly have another column saying what you would do in those circumstances. I’ve tried to incorporate that in some on my funding applications, but word counts are obviously very strict and if people don’t expect it then people aren’t going to put it in. [P021; CI]

One trial statistician described it might be useful to categorise the progression criteria into those that are *fundamental* or *non-fundamental* for progression. However, they shared that this could be challenging as the criteria are often interlinked, e.g. intervention acceptability is likely to correlate with the observed recruitment rate. One trial methodologist stated that there are often *formal* progression criteria that are agreed with the funder and *informal* criteria that are almost like baby steps or things that are running in parallel that researchers ‘keep an eye on’.

### Theme 2: (Avoiding) the potential for personal interest to influence the progression criteria and progression decision-making

Participants often spoke about the potential for personal views or interests of key stakeholders, particularly CIs and PPI representatives, to influence the development of the progression criteria which were more likely to indicate pilot trial ‘success’. Some participants acknowledged that pilot trial progression can be good for a CIs career, creating a temptation to set low or ‘loose’ criteria. Many warned against this. They perceived that although low criteria are more likely to indicate that a pilot trial is feasible, and merit funding for a definitive trial, the assessment of feasibility would likely be inadequate. They shared that this may not truly reflect challenges that might be faced in a definitive RCT and that key questions about trial design would remain unaddressed. One CI highlighted the personal implications of setting loose criteria. On one hand, this may mean a CI is more likely to be awarded further funding which would be good for their career, but on the other hand, running a trial that has progressed inappropriately could result in years of ‘struggling’:

I think historically there’s been a tendency to have too loose a criteria for progression. And this has resulted in trials being moved from pilot through to definitive phase 3 type designs inappropriately. And although that’s good for your career to get this funding, it’s a huge waste of time, money, and effort to do that.[…] I think you have to have an honest conversation with yourself about do you want to spend your next five years struggling through a trial because you failed to answer that question around consent rates. Or withdrawal rates. Or proof of principle. Or acceptability. All of those kind of things. It’s just not an easy way to make a career for yourself. [P021; CI]

Other participants had concerns about CIs setting ‘overly ambitious’ criteria, stating that the progression criteria should be ‘stretching but not so unrealistic that we’re setting ourselves up to fail’.

Just as some participants reported that it could be in the CIs’ interest to see their pilot trial progress, some had similar concerns about PPI involvement. One CI questioned the appropriateness of PPI in discussions around the progression criteria. In their view, PPI representatives are ‘advocates’ for research into a specific area, and personal vested interests in wanting a trial to progress may lead to implicit biases when setting criteria:

If you’re a PPI member, the idea of equipoise, although it’s discussed, it wouldn’t sit naturally. You are an advocate for that condition. And therefore if you’re deciding about what criteria you will use to stop that program of research into a condition that you yourself have been affected by, I think there’s – I don’t want to use the phrase conflict of interest there but it’s kind of heading in that direction. [P021; CI]

One PPI member similarly described how they consider themselves to have an ‘added interest’ in wanting to see research around an illness that they have experienced progress:

Well, I suppose everybody comes with an agenda, whether you like it or not. I’ve had this illness and to see that progress through research to help me is something that I want to be involved with. So I’ve got an added interest. [P033; PPI representative]

Many participants emphasised that this potential for bias could be mitigated by involving a range of stakeholders when developing their progression criteria, including an independent TSC and experienced clinical trials unit (CTU). One trial methodologist described how their CTU offered advice based on their experience to negotiate the progression criteria that were sensible and realistic:

I think if you’ve got a good multidisciplinary team in place and you’re working with a really experienced Trials Unit, you come up with sensible criteria. You come up with a sensible understanding of what the uncertainties might be and you discuss that freely and openly and you’re realistic about it. And a trial team will do that for you. They will pull you up and they will say, “come on, our experience is X, Y and Z. You’re proposing this. That’s far too different to what we’ve seen in similar trials.” So, I do think there’s always a negotiation around what you would expect them to be, but I would like to think they’re the most informed negotiations, rather than just plucking figures out of the air based on nothing. [P032; Trial methodologist]

It was important to some researchers that pilot trials that were not feasible were not considered to have ‘failed’. One CI felt that recognising this might ensure that researchers do not progress pilot trials without sufficient justification, adding that perhaps researchers and funders should consider at a broader level how many pilot trials should be proceeding to definitive trials. This would avoid wasting time and resources associated with doing pilot trials where researchers intend to proceed to a future definitive trial regardless of their findings.

I think if you go into a pilot trial with the mentality that we are going to do the definitive trial regardless, just don’t do the pilot trial. Just go forward and do the definitive trial because otherwise you’re wasting time and resources. [P021; CI]

### Theme 3: Stakeholder engagement in setting the progression criteria and making progression decisions

Whilst participants recognised that involving multiple stakeholders mitigated the potential for personal interests to guide the choice of progression criteria, participants described barriers to involving those stakeholders in practice. The first of these was time. Application deadlines, and other time pressures on CI and stakeholder’s capacity, could mean that feedback on the choice of progression criteria was light touch, or not always forthcoming in time:

I know that our clinical stakeholders would have really important perspective to offer on this and I think they would be interested. It’s just getting their time. Because you can send out a protocol and ‘can you respond in a week?’ and everybody’s really busy so it’s quite hard to get feedback on these things. Then you’re balancing that with just trying to move forward with it. [P019; CI]

Participants also emphasised the importance of involving different stakeholders in progression decision-making. However, time was again a barrier. One CI described that it took months to come to a progression decision that all stakeholders supported:

That takes a bit of time because you’re then looking at the data to say ‘ok well this didn’t work, that didn’t work’ and combining the data with what the trial team say, and then combining the stats with what health economics want, and that takes 3-6 months. But that takes multiple iterations of conversations and then you get a design. [P020; CI]

The second barrier was the lack of information on how to come up with the progression criteria and which stakeholders to involve. This lack of clarity could present difficulties when identifying stakeholder groups, particularly PPI representatives and healthcare providers or the ‘people on the ground’:

There isn’t really any information or there wasn’t about how you would do that, how you would decide it just seemed to be kind of plucked out of the air. [P028; CI]

The third was that the concept of the progression criteria often felt very abstract to lay people who were less familiar with it. These meant that some researchers did not directly discuss the progression criteria with PPI representatives. Whilst others had linked discussions about the pilot trial that indirectly informed progression criteria:

I don’t know that there were discussions specifically around progression criteria. What I think there were, were discussions that linked to progression criteria. So, they were broader discussions about recruitment and challenges with recruitment. Probably indirectly informed the progression criteria but I wouldn’t say directly informed that. [P032; Trial methodologist]

Most PPI representatives did not recall being directly involved in the discussions around the progression criteria, and where PPI were involved sometimes, these discussions were challenging. One PPI representative described attending a meeting to discuss whether the pilot trial would progress, and described talking *round and round the topic* and feeling unsure following the meeting about what decision had been made:

So we had a big meeting just on Monday gone, to decide the way forward and they talked round and round and round the topic and I’m not sure, even though I was there, I’m not sure what they’ve actually decided […] They’re looking at it as traffic lights, and stop and go, and minor surgery, and major surgery, and these are all new terms for me completely. [P017; PPI representative]

Involving independent TSCs acting on behalf of research funders to approve the progression criteria also offers the potential to mitigate bias. However, in reality, this presented a causality dilemma for some CIs, as the progression criteria were required in the funding application, which was developed before they had convened a TSC. As a result, often the criteria stipulated in funding applications had not been reviewed by a TSC, nor had input from other stakeholders, e.g. trial managers who were appointed once funding had been awarded. Some researchers also suggested that a lack of direct dialogue with funders could limit the choice of progression criteria, with researchers keen to deliver funding applications that are likely to be successful. Participants who were affiliated to research funding panels described how panellists often reflect positively on the inclusion of clear progression criteria and that their inclusion would likely lead to a higher scored application:

If it’s got clear feasibility progression criteria, sure, I would score it higher. It gives it that extra quality in my view. [P007; Trial statistician and methodologist]

### Theme 4: Lessons learned from doing the pilot trial and their impact on progression criteria applicability

Many participants perceived pilot trials as opportunities to learn. Researchers described making small adaptations to the pilot trial design throughout its conduct in response to unanticipated events, or lessons that they had learned whilst running the pilot. Some researchers drew parallels between this aspect and that of an adaptive trial design. One CI described how they had used their qualitative research findings to refine and update the intervention and trial processes throughout the pilot trial, meaning that the pilot trial they ended up with looked quite different to their initial design:

The qualitative work was actually used to refine the intervention and the trial processes that were delivered as we went along[…] What we ended up with was a trial design and an intervention that was slightly different to what we started with[…] If we went to full trial, it would be more closely aligned to what we finished with than what we started with. [P004; CI]

Learning from, and responding to, unanticipated events could mean that some progression criteria are no longer applicable. There was inconsistency in how participants thought this should be managed. One CI described that changing how they delivered their intervention meant that their fidelity progression criteria were no longer applicable. Following discussion with their TSC, this CI decided to keep the progression criteria but alter the way that it was defined so that fidelity was assessed in a different way. Most statisticians did not support changing the progression criteria if they were no longer relevant; one suggested instead that researchers report transparent and honest reasons for why their criteria were no longer applicable:

I’m not sure that changing the criteria is the best way to go about it, I think what you want to do is abandon your criteria at the end of the trial and to give like really transparent honest reasons about why you don’t think its applicable anymore. [P003; Trial statistician and methodologist]

### Theme 5: Other factors that inform the progression decision

Participants were conflicted on the extent to which they relied on the progression criteria to inform decisions about feasibility. Where participants described pilot trials that went ‘really well’, often all of the progression criteria had been met and they considered feasibility to be clear or ‘obvious’. However, this was not true for all, with some participants describing situations where all the progression criteria were met, yet their pilot trial was not feasible in practice. In the more likely scenario where a pilot trial meets some, but not all, of their progression criteria progression decisions were more complex. One CI shared that his advice to other researchers would be to think in detail about how they would handle the ‘very realistic eventuality’ that they meet some but not all of their progression criteria and prepare for it rather than regarding it as an ‘unlikely or worst-case scenario’. One trial methodologist described that whilst the progression criteria might present a ‘nice algorithm’ to help make a progression decision, in practice, this is not always as straightforward.

I think if anything, the trial showed that yeah you can have all the criteria, but it still may not be something that’s worth continuing with if you have other factors. [P012; Trial Manager]

Signals of efficacy, qualitative findings, continuity of the research team and enthusiasm for the research were also key drivers of pilot trial progression.

Although many participants shared that observed signals of efficacy can impact progression decisions, they were conflicted about the direction of this impact. Some thought that pilot trials were more likely to progress if they demonstrate a signal of efficacy (as a subsequent trial showed promise), yet one trial statistician shared that demonstrating a signal of efficacy deterred progression, with funders considering that a definitive trial was no longer needed since the pilot trial had already demonstrated an effect.

We’d just tickled statistical significant difference. So it was small numbers. So one of the stats guys was like well I hope you’re not deciding to progress to try and apply for money just because it looks like it might work. Which we weren’t, but that was something that people had said oh that’s interesting. [P011; CI]

One[trial had] quite a large sample size for a pilot study and we showed actually a between-group difference. Even though statistically speaking we shouldn’t be doing that or it’s not what anybody wants to see, and we had veered away from showing the P-values or anything so it was kind of like confidence interval for between-group difference, the confidence interval was greater than 0, so you know that kind of stopped it a little bit and the funders were a bit uneasy because it’s almost as if you’ve shown effect already. [P007; Trial statistician and methodologist]

Most participants emphasised the importance of drawing on qualitative findings to help ‘build a picture’ and provide explanation and context for whether progression criteria were or were not met. One CI described how their qualitative findings conflicted with their progression criteria findings, demonstrating the value to this researcher of conducting qualitative research within their pilot trial.

The intervention was obviously not quite right. And the progression criteria didn’t pick that up, actually.[…] I had never really thought about that, actually, but progression criteria alone wouldn’t have picked that up if we hadn’t done the qualitative work. [P028; CI]

Some trial statisticians suggested that qualitative interpretations of feasibility might be more appropriate than relying on the progression criteria when interpreting the pilot trial findings, since there are lots of ‘contextual factors’ that do not easily fit into simple metrics.

So I’m going to say something you wouldn’t expect a statistician to say, I think it’s very hard to quantify what you learn from a pilot study. And there’s a lot of contextual factors that don’t equate neatly into usual metrics. [P029; Trial Statistician]

Many participants described team continuity and continued enthusiasm were important drivers of progression. Researchers expressed that maintaining enthusiasm can be challenging, particularly during the pause between the pilot and definitive RCT. Some CIs described losing trial managers to other studies, and other CIs described expectations that they return to clinical work following fellowships. These external pressures and competing priorities can result in changes to the research team and mean that the *people knowledge* and enthusiasm developed throughout the pilot trial is lost.

The people on the study are the clinical people, and if you have a gap your trial manager leaves and so on, and you’re starting again a little bit. There’s a bit of knowledge but it’s mainly people knowledge I would say. [P018; CI]

As discussed previously in the third theme, multi-stakeholder involvement in progression decision-making was also crucial but presented complex challenges in practice.

### Theme 6: Progression of external randomised pilot trials—funding considerations and constraints

Researchers described funding-related challenges for pilot trials and their progression. Conducting pilot trials on limited budgets, an inability to obtain further funding, and the length of time associated with obtaining further funding can compromise the pilot trial progression.

Many researchers shared that limited funding can mean that delivering pilot trials to budget can come at a cost to trial design. As described in theme three, participants value a multi-stakeholder approach to setting their progression criteria, yet participants who had trial oversight roles described that covering the costs of pilot trial personnel and support, such as a CTU, qualitative researcher or health economist within their budget was challenging.

It’s harder for CTUs to support RfPB now because the funding is so small, and the funding threshold hasn’t gone up in light of salaries going up and every other inflation so we are increasingly having to turn investigators away and say we can’t work on the RfPB because you basically can’t afford a CTU or if we’re doing it were doing it at a loss and then that becomes unsustainable. [P010; Trial Manager]

Some researchers described delivering pilot trials on a ‘shoestring’ budget with the pilot trial design ‘dictated by the cost-constraints’. Some CIs described that including the cost of an embedded qualitative research study within their pilot trial was challenging, even though as described in theme four, many researchers perceived qualitative findings to be an important consideration of pilot trial progression. One trial methodologist described using short-term outcomes in their pilot trial even though long-term outcomes were planned for the full trial because it was ‘probably too expensive to do a 12-month follow-up’.

As described in theme 1, some participants considered further funding to be their ultimate progression criteria, and many described challenges with obtaining further funding following their pilot trial, even if all progression criteria were met. One trial statistician offered a potential reason for this problem, stating from their perspective there seemed to be more funding available for external pilot trials than for definitive trials:

I’m not saying it’s causal, but I think the way that funding mechanisms are set up does incentivise certain types of research behaviour. Researchers are a little bit chase-the-money. And RfPB and CSO in Scotland and their funding envelopes have set up a situation where it’s quite acceptable and lucrative to apply for external pilots on a regular basis […]. There is a supply and demand issue that there’s probably more demand for funding successful external pilots than there might be funding to do definitive trials. So, there’s a bottleneck. [P025; Trial statistician and senior researcher]

This conflict between feasibility and progression can result in research waste. One CI described their frustration with this situation:

You can do a brilliant pilot study and everything goes absolutely to plan; you recruit, you show likely effectiveness and cost effectiveness and it still doesn’t get funded for the full study. So, this is a big disappointment. [P031; CI]

Many researchers described facing significant delays between pilot and definitive trial whilst they published their pilot trial findings and prepared a definitive trial funding application. Researchers considered the amount of time that an external pilot trial adds to the research pathway to be a limitation, as researchers move on to other things and the healthcare landscape swiftly changes:

I do think it presents a barrier and I think there’s a risk that you end up in a sort of no-man’s land and then, as is the nature of research, you get swept away with other things. [P032; Trial methodologist]

One CI said that as they approach the end of their career, they no longer have the time to do an external pilot and full trial and would now opt to do an internal pilot trial to speed up the research process.

To avoid this scenario, some researchers expressed that they would like a more streamlined route of progression from external pilot to definitive RCT. Some suggested that there could be an ‘understanding’ with funders that if the pilot trial goes well there is a good chance that a definitive trial would be funded. Others suggested that the funding process should be sped up or fast tracked, so there is a shorter time frame between an external pilot finishing and definitive RCT starting. Some described that if there were minimal changes made during the pilot trial, there’s a benefit to funders (as well as researchers and participants) in allowing an external pilot trial to transition more seamlessly into a definitive RCT, resembling the design of an internal pilot trial upon completion. Some researchers described how in exceptional cases certain funders had facilitated this streamlined progression, but that this was far from the norm.

You want to be able to write it as an external that you can go straight in and carry on. And that you have a checkpoint and then you get the rest of the funding. Definitely. [P026; Trial methodologist and senior researcher]

### Overarching theme—a one-size approach to progression does not fit all

Underpinning all themes was the highly context-dependent nature of external pilot trials. We therefore developed the overarching theme, ‘a one-size approach to progression does not fit all’, to illustrate this broader understanding. We found that there is no one approach that fits all trials, and trial teams, for the development of the progression criteria and progression decision making. This was illustrated by one participant who outlined the importance of leaving some room for context:

It’s not a one size fits all, so there’s always a bit of room for a… or there needs to be some room for context. [P031; CI]

For many researchers, progression decisions were based on more than meeting their progression criteria. The progression decision was also influenced by unanticipated events during the pilot trial (i.e. are internal to the pilot trial but were not pre-empted) such as challenges with delivering the intervention, qualitative research findings, signals of efficacy, and factors outside (i.e. external to) the pilot trial such changes to the setting, trials competing for the same population, continuity of the research team and availability of definitive trial funding. Participants articulated a preference towards the more flexible stop-amend-go format to progression criteria, to accommodate the contextual nature of external pilot trials, and acknowledge that progression decision-making is ‘not black and white’.

The members of the pilot trial research teams can also have different views on the importance of the progression criteria when interpreting pilot trial findings. This can mean that reaching a conclusion about feasibility that all researchers agree on can be challenging, as ‘feasibility’ of the same pilot trial may be interpreted differently by stakeholders who value the progression criteria to different extents. Some researchers described that ultimately progression is dependent on ‘whether you can make a credible case to your funder that they should invest in this next stage’. However, researchers shared varied experiences to indicate that there is no guarantee that meeting all progression criteria will result in a successful funding award, implying that external factors exist within the wider funding landscape that also have a bearing on pilot trial progression.

## Discussion

### Summary of findings

This study presents key stakeholders’ experiences and perspectives of making progression decisions following external randomised pilot trials, and how progression criteria inform these decisions. Researchers had mixed perceptions of the value of the progression criteria and described varied ways in which they had been developed. Researchers suggested that a multi-stakeholder approach to setting progression criteria and making progression decisions can help avoid biases. However, researchers described several other factors that informed their progression decisions. These include looking for signals of efficacy, qualitative interpretations of feasibility, continuation of the research team and enthusiasm for the research.

For many researchers, the ultimate barrier to pilot trial progression was whether further funding could be obtained, even if all the criteria were met. We developed an overarching theme to encompass our findings: a one-size approach to progression does not fit all. No two external pilot trials are the same, nor is the context in which they are implemented. Our findings highlight some of the contextual considerations, e.g. around research funding, trial teams and enthusiasm for the research, which can present both facilitators and barriers to pilot trial progression.

### Findings in context

Many recommendations proposed by Avery et al. (2017) for using progression criteria in internal pilot trials [2] were also followed for external pilot trials, including the stop-amend-go approach and striking a balance between flexibility and firmness. However, some were not met as easily, for example, the agreeing and assessing criteria with an independent oversight committee. Researchers highlighted a lack of knowledge on how they should be developing their progression criteria, who should be involved and how best to do so. Many suggested that more guidance and resources would be beneficial to address this. Our research also highlighted inconsistencies between researcher and PPI representative experiences. Researchers were conflicted on whether, and how, to involve PPI in key decisions, with many suggesting that they are involved through their roles on TMGs and TSCs. However, PPI representatives rarely reported their perspectives on how they had contributed to these discussions. Future research could explore in more detail the role of PPI in external pilot trials.

The CONSORT extension to randomised pilot and feasibility trials [19] states that researchers should report ‘if applicable, prespecified criteria used to judge whether, or how, to proceed with future definitive trial’ (Item 6c). This suggests that there might be instances where the progression criteria are not applicable, but the circumstances for this are unclear. We found that some researchers considered the progression criteria to not be applicable where they were not informed by existing data. Others considered criteria to be altogether more applicable to internal pilot trials where meeting them would almost certainly result in progression to the main RCT phase, for which funding had already been awarded. Whereas for external pilot trials meeting the progression criteria do not necessarily guarantee progression, for which funding is often the ultimate barrier. This supports previous research which identified the potential for wasted resources and opportunities where feasibility studies that were considered to be feasible do not proceed to future research [20]. This begs the question: are we doing too many external randomised pilot trials?

Many researchers emphasised the benefit of doing qualitative research in pilot trials and considering qualitative findings when making progression decisions, including drawing on their own experiences and lessons learned from doing the pilot trial. This is in line with previous guidance to identify and appraise potential solutions to problems that were faced during the pilot trial, to facilitate transparent decision-making [21]. O’Cathain et al. outline a number of questions that feasibility studies might have that could be met using qualitative research methods [22]. Yet, a previous meta-epidemiological study found that researchers do not often include qualitative research methods in their pilot trial design [23]. Our findings suggest that this could often be due to the challenges of embedding qualitative research in pilot trials with limited funding.

### Strengths and limitations

A key strength of this study is the inclusion of a diverse sample of participants with a broad range of skills and experiences. A limitation is that we only included participants who were based in the UK and have not explored whether there are international differences in approaches to progression criteria and progression decision-making. A further limitation is that we were unable to fully explore the role of PPI in pilot trials. We also did not seek PPI involvement in the design of this study, and in hindsight, PPI review of the PPI interview schedule would have been valuable. PPI representatives were particularly difficult to sample, and so we suggest that the views and experiences of PPI representatives in external pilot trials are an area for further research which would require more time and focused attention.

### Future research and interim recommendations

Pre-specified progression criteria aim to avoid bias and promote transparent pilot trial progression decision-making so that pilot trials only progress where a future trial is truly considered feasible. Although the progression criteria for external pilot trials are required by some research funders [24] and their reporting in pilot trial publications is encouraged [19], our findings highlight the need to develop clear evidence-based recommendations for researchers for determining pilot trial feasibility.

Based on these findings, we suggest researchers think about the progression criteria from the earliest opportunity and consider what is appropriate for their specific pilot trial in its specific context given the feasibility objectives. Researchers should have some rationale or justification for their choice of progression criteria, e.g. findings of related research studies, clinical observations or audit. We encourage innovative ways of thinking about the progression criteria as a method of feasibility assessment, for example, how best to integrate qualitative research findings. Our findings have identified the benefit of engaging various stakeholders in discussions around the progression criteria, including wider trial teams, PPI representatives, clinical colleagues (particularly those who will be implementing the trial), funders and seeking the input of Research Design Services. Future development of resources to explain what the progression criteria are, why they are important and best practices for developing the progression criteria (such as who to engage and how) would facilitate these discussions. Finally, we encourage researchers to recognise that feasibility and progression are two separate decisions. Pilot trials might be feasible but might not progress, yet these contextual considerations that underpin progression decisions might go undocumented and unreported. The best way to address this is an important question that requires further consideration.

## Conclusions

A one-size approach does not fit all when it comes to the progression criteria and progression decision-making, due to the unique uncertainties and unique contexts of individual pilot trials and the settings in which they are implemented. Participants reported differing views and processes in how the progression criteria were developed. This could result in setting inappropriate progression criteria that are biased by personal interests of key stakeholders or reusing criteria from other trials without careful consideration of their applicability.

Our findings also highlight the preference amongst some researchers towards qualitative interpretations of feasibility, drawing on lessons learned and experience from doing the pilot trial itself. This could indicate that the shift towards framing pilot trial findings around formal prespecified progression criteria restricts how researchers consider, interpret and determine feasibility, with an increased focus on addressing anticipated problems and less learning from unanticipated challenges faced. Signals of efficacy, qualitative findings, continuation of the research team, enthusiasm for the trial and stakeholders’ perspectives can also inform progression decision-making, with the key factor being the availability of funding for a future definitive trial.

## Supplementary Information

**Additional file 1.** Interview topic guides.

**Additional file 2.** Checklist of trustworthiness.

## Data Availability

De-identified framework matrices and the final version of the developed analytical framework are available from the corresponding author on reasonable request. Informed consent forms are available on request. Interview schedules are presented in Additional file 1.

## Abbreviations

CI: Clinical investigator
CONSORT: Consolidated Standards of Reporting Trials
CSO: Chief Scientist Office
CTU: Clinical Trials Unit
DPA: Data Protection Agreement
HTA: Health Technology Assessment
NIHR: National Institute for Health Research
PAFS: Pilot and Feasibility Studies
PIS: Participant information sheet
PPI: Patient and public involvement
RCT: Randomised controlled trial
RDS: Research Design Service
RfPB: Research for Patient Benefit
TM: Trial manager
TMRP: Trials Methodology Research Partnership
TSC: Trial Steering Committee
UK: UK
UKCRC: UK Clinical Research Collaboration
UKTMN: UK Trial Managers’ Network

## References

[1] Eldridge SM, Lancaster GA, Campbell MJ, Thabane L, Hopewell S, Coleman CL, et al. Defining feasibility and pilot studies in preparation for randomised controlled trials: development of a conceptual framework. Lazzeri C, editor. PLoS ONE; 2016;11:e0150205. Available from: 10.1371/journal.pone.0150205 10.1371/journal.pone.0150205 PMC4792418 26978655

[2] Avery KNL, Williamson PR, Gamble C, Francischetto EOC, Metcalfe C, Davidson P (2017). Informing efficient randomised controlled trials: exploration of challenges in developing progression criteria for internal pilot studies. BMJ Open..

[3] Mellor K, Eddy S, Peckham N, Bond CM, Campbell MJ, Lancaster GA (2021). Progression from external pilot to definitive randomised controlled trial: a methodological review of progression criteria reporting. BMJ Open.

[4] Mellor K, Albury C, Hopewell S. Using progression criteria to determine feasibility of external randomised pilot trials: protocol for a qualitative study of stakeholder views. OSF. 2020; Available from: https://osf.io/5n2kz.

[5] Tong A, Sainsbury P, Craig J (2007). Consolidated criteria for reporting qualitative research (COREQ): a 32-item checklist for interviews and focus groups. Int J Qual Heal Care.

[6] Corbin, J. & Strauss, A. Theoretical sampling. In Basics of qualitative research (3rd ed.): Techniques and procedures for developing grounded theory. SAGE Publications, Inc.; 2008. pp. 143-158. 10.4135/9781452230153.

[7] Palinkas LA, Horwitz SM, Green CA, Wisdom JP, Duan N, Hoagwood K. Purposeful sampling for qualitative data collection and analysis in mixed method implementation research. Adm Policy Ment Heal Ment Heal Serv Res. Springer New York LLC; 2015;42:533–544. 10.1007/s10488-013-0528-y PMC4012002 24193818

[8] UKCRC (2020). UK Clinical Research Collaboration.

[9] Network Hubs (2020). TMRP.

[10] About the UKTMN (2020). UKTMN.

[11] Research Design Service (2020). NIHR.

[12] Vasileiou K, Barnett J, Thorpe S, Young T (2018). Characterising and justifying sample size sufficiency in interview-based studies: systematic analysis of qualitative health research over a 15-year period. BMC Med Res Methodol.

[13] McGrath C, Palmgren PJ, Liljedahl M (2018). Twelve tips for conducting qualitative research interviews. Taylor & Francis.

[14] Gale NK, Heath G, Cameron E, Rashid S, Redwood S (2013). Using the framework method for the analysis of qualitative data in multi-disciplinary health research. BMC Med Res Methodol.

[15] Braun V, Clarke V (2019). Reflecting on reflexive thematic analysis. Qual. Res. Sport. Exerc. Heal. Routledge.

[16] Lincoln YS, Guba EG. Naturalistic inquiry. Newbury Park, CA: Sage Publications; 1985 . Available from: https://books.google.co.uk/books?hl=en&lr=&id=2oA9aWlNeooC&oi=fnd&pg=PA5&sig=GoKaBo0eIoPy4qeqRyuozZo1CqM&dq=naturalistic+inquiry&prev=http://scholar.google.com/scholar%3Fq%3Dnaturalistic%2Binquiry%26num%3D100%26hl%3Den%26lr%3D&redir_esc=y#v=onepage&q=naturalistic inquiry&f=false

[17] Cohen D, Crabtree B. Qualitative Research Guidelines Project. 2006. Available from: http://www.qualres.org/index.html.

[18] Hanson CS, Ju A, Tong A. Appraisal of qualitative studies. Handb Res Methods Heal Soc Sci. Springer Singapore; 2019. 1013–1026. Available from: 10.1007/978-981-10-5251-4_119

[19] Eldridge SM, Chan CL, Campbell MJ, Bond CM, Hopewell S, Thabane L (2016). CONSORT 2010 statement: extension to randomised pilot and feasibility trials. BMJ.

[20] Morgan B, Hejdenberg J, Hinrichs-Krapels S, Armstrong D (2018). Do feasibility studies contribute to, or avoid, waste in research. PLoS One.

[21] Bugge C, Williams B, Hagen S, Logan J, Glazener C, Pringle S, Sinclair L (2013). A process for Decision-making after Pilot and feasibility Trials (ADePT): development following a feasibility study of a complex intervention for pelvic organ prolapse. Trials [Internet]. Trials.

[22] O’Cathain A, Hoddinott P, Lewin S, Thomas KJ, Young B, Adamson J (2015). Maximising the impact of qualitative research in feasibility studies for randomised controlled trials: guidance for researchers. Pilot Feasibility Stud.

[23] Baldeh T, MacDonald T, Kosa SD, Lawson DO, Stalteri R, Olaiya OR (2020). More pilot trials could plan to use qualitative data: a meta-epidemiological study. Pilot Feasibility Stud.

[24] NIHR (2021). Guidance on applying for feasibility studies.

