# Supplementary material for: How are progression decisions made following external randomised pilot trials? A qualitative interview study and framework analysis
Source: Trials. 2022 Feb 10;23:132. doi: 10.1186/s13063-022-06063-9 (PMC8832640; doi:10.1186/s13063-022-06063-9)
Supplement: Supplementary file 1 — Additional file 1. Interview topic guides. [file 13063_2022_6063_MOESM1_ESM.docx]

**SUPPLEMENTARY FILE 1** Interview topic guides

Whilst we will address the questions below during the interview, the qualitative work will remain flexible, allowing participants to guide the conversations and present what is important to them. It is best-practice during qualitative interview projects to iteratively develop topics and questions as new ideas are identified from early data collection. Therefore, following best-practice, we may add new topics as the interviews progress and data collection continues. The key focus of these interviews will be researchers’ experience with, and thoughts about, using progression criteria to inform the decision to progress from a pilot Randomised Controlled Trial (RCT) to a definitive RCT.

**Interview guide 1: RESEARCHER**

**QUESTIONS AND PROMPTS**

1. **Can you tell me about your current role in relation to external randomised pilot trials?**
   1. What institution?
   2. For how long?
   3. Involvement with a range or very few clinical trials and pilot trials?
   4. Do you contribute towards a NIHR Research Design Service?
   5. Do you contribute towards any funding committees?
   6. Any journal affiliations e.g. editor, peer reviewer?
2. **Can you tell me about your experience of applying for external pilot trial funding?**
   1. Which funding steams?
   2. How would you describe the application process?
   3. Were there any challenges?
   4. Was funding obtained?
   5. Do you have a lot of experience with these funding applications?
3. **Can you tell me a bit more about the external randomised pilot trial(s) you have been involved with?**
   1. What was the aim of the trial?
   2. Why was the trial done/what were the uncertainties about feasibility?
   3. What were the findings?
      1. Did you decide to proceed/proceed with changes/not proceed etc
4. **What were the feasibility outcomes?**
   1. Why were they important?
   2. Were they typical to other randomised pilot trials you’ve been involved with?
5. **Did the pilot trial(s) have a qualitative research component?**
   1. What was the aim of the qualitative research?
6. **Did the pilot trial(s) have any Patient and Public Involvement?**
   1. How was PPI used?
7. **Did the pilot trial(s) have any progression criteria?**
   1. You might have used a different term to describe “progression criteria”
      ****IF NO – USE ALTERNATIVE QUESTIONS****
8. **Can you describe the progression criteria used in the pilot trial(s)?**
9. What feasibility outcomes were progression criteria based on?
10. What were the specific targets?
11. Was a traffic light system used?
12. **How did you establish the progression criteria?**
13. Why were these criteria important to you?
14. How did you decide on specific targets?
15. Did you follow any guidance?
16. Who was involved?
    1. Trial steering committee?
    2. Trial management group?
    3. Funders?
    4. Patient and public representatives (if applicable)?
17. **Did the trial(s) meet the progression criteria?**
    1. All? Some?
18. **How did you assess the progression criteria at the end of the trial(s)?**
19. Who was involved?
    1. Funders?
    2. TSC?
    3. PPI?
20. **How did you decide whether a future trial was feasible?**
    1. How/did progression criteria help you make this decision?
    2. Were there other things you considered in addition to the progression criteria?
    3. Will you make changes to the future/definitive trial?
21. Who was involved in making this decision?
22. **Were there any challenges in relation to using progression criteria?**
23. Can you give examples?
24. Were there any challenges with designing progression criteria?
25. Were there any challenges with assessing progression criteria?
    1. Where not all targets are met?
26. Did the progression criteria change during the pilot trial?
27. If you could run the trial again, would you do anything differently?
28. **What advice would you give to someone who was planning to do an external pilot trial, who not previously used progression criteria?**
29. Will you use progression criteria in the future?
30. Why do you think that is important? What is the benefit?
31. Did that work well for you?
32. Will you make this change going forward?

****ALTERNATIVE QUESTIONS FOR PARTICIPANTS WHO DID NOT USE PROGRESSION CRITERIA****

1. **Were you aware of progression criteria but decided not to use it?**
   1. If yes, why?
   2. Did you face any challenges?
2. **How did you decide whether a future trial was feasible?**
   1. What outcomes was this decision based on?
      1. Were there any other things you considered?
   2. Who was involved in making this decision?
      1. Funders?
      2. TSC?
      3. PPI?
   3. If feasible, will you make any changes to the future trial?
3. **Would you consider using progression criteria in external pilot trials you are involved with going forward?**
   1. What do you think the benefit would be?
   2. Would you look for support or guidance in using progression criteria?
      1. If so, where would you search?
   3. Who would you involve in discussion around what progression criteria to use?

**Interview guide 2: PPI REPRESENATIVE**

**QUESTIONS AND PROMPTS**

1. **Can you please tell me a little about your role as a participant representative?**
   1. What does your role entail?
   2. How long have you been a participant representative for?
   3. How many clinical trials have you contributed to?
   4. How many were external pilot trials?
2. **Can you tell me about the pilot trial/s you have been involved in?**
   1. How did you get involved as a participant representative?
   2. What was the pilot trial trying to find out?
3. **Were there any other participant representatives for the pilot trial?**
4. **Were there any challenges?**
   1. Is this the same for other trials you’ve contributed to e.g. non pilot trials?
5. **What were the findings of the external pilot trial?**
   1. Did you decide to proceed/proceed with changes/not proceed etc
   2. How was this decision made?
   3. [If proceed] – Will you be involved as a participant representative in the future trial?
6. **What is important to you when deciding whether researchers should do a future trial after this one?**
   1. What do you mean by [……]?
   2. Can you give an example?
   3. Why is this important to you?
7. **Was there any qualitative research e.g. interviewing patients about being in the trial?**
   1. How important is this for pilot trials?
8. **Do you recall discussing progression criteria?**
   1. Is this a familiar term?
   2. You might have used a different term to describe “progression criteria”

****IF NO – USE ALTERNATIVE QUESTIONS****

1. **Can you describe the progression criteria that were used?**
   1. What were progression criteria based on?
   2. What were the specific targets?
   3. Was a traffic light system used?
2. **Were you involved in establishing these progression criteria when the trial was being designed at the start?**
   1. Who else was involved?
3. **Were you involved in discussing the progression criteria when the trial finished?**
   1. Did you meet all or some of the progression criteria?
   2. Who else was involved?
   3. Did progression criteria help the team decide whether to do the future trial?
   4. Did you discuss whether the future trial design would change based on what happened in the pilot trial?
4. **How important do you think it is that participant representatives like yourself are involved in this process?**
   1. Establishing progression criteria at the start?
   2. Assessing progression criteria at the end?
   3. Can you give examples to demonstrate what you’ve mentioned?
5. **Can you tell me whether any part of the process was challenging?**
   1. Can you give examples to demonstrate what you’ve mentioned?
   2. Was progression criteria a new concept?
   3. Challenges with establishing progression criteria at the start?
   4. Challenges with assessing progression criteria at the end?
6. **Is there anything that you have since reflected on about the progression criteria used, that perhaps you did not think of or say to the research team at the time?**
   1. Can you give examples to demonstrate what you’ve mentioned?
   2. Would this have changed the progression criteria?

****ALTERNATIVE QUESTIONS FOR PARTICIPANTS WHO ARE NOT FAMILIAR WITH PROGRESSION CRITERIA****

***Explanation:*** Progression criteria are targets that researchers set at the start of a pilot trial, for example to recruit at least X participants and to retain at least Y. Researchers then look at these targets at the end of the trial to help them decide whether they should do the future trial and if it is likely to work.

1. **How important do you think it is that participant representatives are involved in setting these targets at the start of the trial?**
   1. Can you give examples to demonstrate what you’ve mentioned?
   2. What do you think the benefits could be?
2. **How important do you think it is that participant representatives are involved in looking at these targets at the end of the trial to say whether or not we should do the future trial?**
   1. Can you give examples to demonstrate what you’ve mentioned?
   2. What do you think the benefits could be?
3. **Do you think there would be any challenges of being involved in these discussions about progression criteria?**
4. Before hearing about this research, was progression criteria a new concept?
5. Can you give examples to demonstrate what you’ve mentioned?
6. What challenges might there be in designing the progression criteria?
7. What challenges might there be in assessing the progression criteria?
