# Supplementary material for: How are progression decisions made following external randomised pilot trials? A qualitative interview study and framework analysis
Source: Trials. 2022 Feb 10;23:132. doi: 10.1186/s13063-022-06063-9 (PMC8832640; doi:10.1186/s13063-022-06063-9)
Supplement: Supplementary file 2 — Additional file 2. Checklist of trustworthiness. [file 13063_2022_6063_MOESM2_ESM.docx]

**SUPPLEMENTARY FILE 2** Checklist of trustworthiness

| **Criteria** | **Description** | **Achieved** | **Techniques used** |
| --- | --- | --- | --- |
| **Credibility** (Internal validity) | Based on the principle of truth or reality.  *Are the findings trusted? Do the findings provide a comprehensive and sensible interpretation of the data?* | **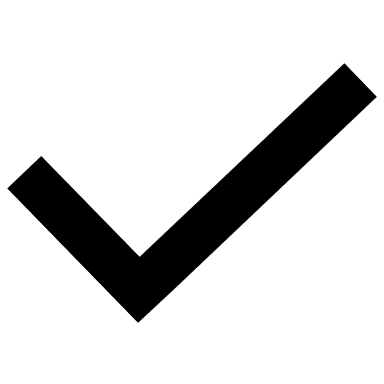** | - **Triangulation**: The findings are triangulated with other research that has been conducted both by our group and is published in the wider literature. - **Peer discussion**: Segments of coded transcripts and the analytical framework, developed by KM, was peer discussed with an experienced qualitative researcher (CA), to acknowledge that qualitative research data is *co-created* since different researchers place value on different things. Engaging in peer discussion during data analysis allows researchers to challenge their presumptions. - **Respondent validation**: All participants were invited to participate in a respondent validation (or “member checking”) exercise where they were sent a short summary of their interview transcript and given the opportunity to comment on how well they thought this summarised the interview and add any further comments. |
| **Dependability** (Reliability) | Based on the principle of consistency.  *Is the research process transparent? Is the research auditable?* | **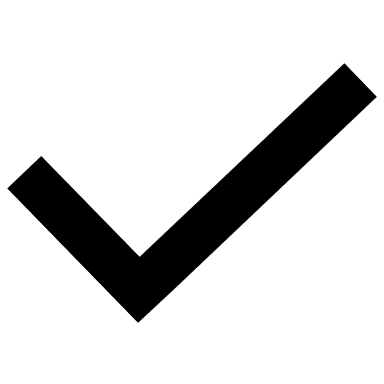** | - **Transparent reporting**: The methods and findings are reported in line with the COREQ guidelines for reporting qualitative research. - **Audio-recording**: Interviews were audio-recorded using two Dictaphones. - **Transcription**: All interviews were transcribed by the KM or an experienced transcription service. All transcripts were checked for errors by re-listening to the audio-recording. - **Computer software**: NVivo (v12) software was used to store and organise data and facilitate coding. - **Audit trail**: An audit trail was kept to document decisions made during the research study. Audit documentation included key questions documented in the reflexivity journal when being reflexive, project meeting minutes, and a record of memo’s created within NVivo (v12) to draw attention to sections of the data that were of particular interest to the researcher during coding. The audit trail was reviewed during later analytical stages of data analysis to supplement interpretation of the findings and guide theoretical sampling. |
| **Transferability** (External validity) | Based on the principle of applicability or relevance.  *Are the findings relevant or applicable to other contexts?* | **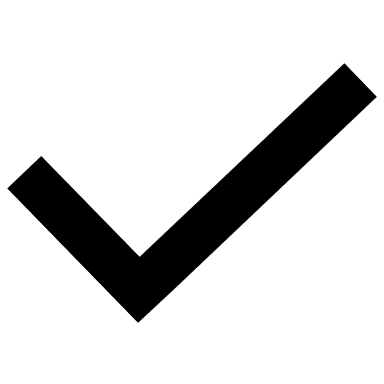** | - **Thick description**: The sample and methods used are described in detail to allow other researchers to evaluate whether the findings might be relevant to other contexts or settings. |
| **Confirmability** (Objectivity) | Based on the principle of neutrality.  *Do the findings reflect the perspectives of the participants and are linked to the data?* | **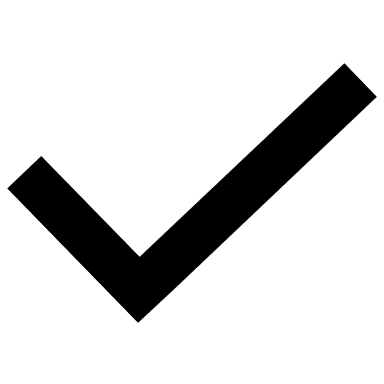** | - **Reflexivity**: KM documented reflections in a reflexivity diary throughout the study. Maintaining a reflexivity diary allowed KM to recognise and challenge any implicit biases she had. The reflexivity diary was also important in guiding theoretical sampling decisions. - **Inclusion of raw data**: Verbatim data was highlighted in the framework matrices and direct quotes are reported. |
